# Robust Automatic Target Recognition via HRRP Sequence Based on Scatterer Matching

**DOI:** 10.3390/s18020593

**Published:** 2018-02-14

**Authors:** Yuan Jiang, Yang Li, Jinjian Cai, Yanhua Wang, Jia Xu

**Affiliations:** 1 Radar Research Lab, School of Information and Electronics, Beijing Institute of Technology, Beijing 100081, China; sarah900122@163.com (Y.J.); liyang@racobit.com (Y.L.); cjjbit@163.com (J.C.); xujia@bit.edu.cn (J.X.); 2 Beijing Key Laboratory of Embedded Real-time Information Processing Technology, Beijing Institute of Technology, Beijing 100081, China

**Keywords:** automatic target recognition (ATR), high resolution range profile (HRRP), singular value decomposition (SVD), atomic norm minimization (ANM), feature extraction

## Abstract

High resolution range profile (HRRP) plays an important role in wideband radar automatic target recognition (ATR). In order to alleviate the sensitivity to clutter and target aspect, employing a sequence of HRRP is a promising approach to enhance the ATR performance. In this paper, a novel HRRP sequence-matching method based on singular value decomposition (SVD) is proposed. First, the HRRP sequence is decoupled into the angle space and the range space via SVD, which correspond to the span of the left and the right singular vectors, respectively. Second, atomic norm minimization (ANM) is utilized to estimate dominant scatterers in the range space and the Hausdorff distance is employed to measure the scatter similarity between the test and training data. Next, the angle space similarity between the test and training data is evaluated based on the left singular vector correlations. Finally, the range space matching result and the angle space correlation are fused with the singular values as weights. Simulation and outfield experimental results demonstrate that the proposed matching metric is a robust similarity measure for HRRP sequence recognition.

## 1. Introduction

High resolution range profile (HRRP)-based automatic target recognition (ATR) has drawn much attention for wideband radars [1,2,3,4,5,6]. As the one-dimensional high-resolution ‘image’ of a target, HRRP can effectively reflect energy and structure information of multiple scatterers for a distributed target along the slant range direction with respect to a certain radar line-of-sight (RLOS). Furthermore, compared with the two-dimensional high-resolution synthetic aperture radar (SAR) or inverse synthetic aperture radar (ISAR) imaging [7,8,9], HRRP-based ATR has a lower requirement on the RLOS change, lower system complexity and higher computational efficiency. Effective HRRP features and feature extraction approaches have been researched in the literature [10,11,12,13,14]. Although the structure features have excellent performances in HRRP-based ATR, they still mainly face two problems of background interference and aspect sensitivity. 

The background noise will inevitably affect the performance for ATR [12,13,14]. Specifically, the training data is usually cooperatively collected in an ideal environment, while the test data is collected non-cooperatively with sheltering of interference, which requires the feature extraction to be robust for ATR in both high signal-to-noise ratio (SNR) and low SNR scenarios. To improve the robustness, structure features including the high-order spectrums and dominant scatterers are explored [15,16,17,18,19]. Compared to the high-order spectrum features with high dimensions, the scatterers are much easier for extraction with a lower computation requirement. In [19], the dominant scatterers are extracted over the Fourier dictionary with an orthogonal matching pursuit (OMP) algorithm based on a single HRRP, whose performance is affected by the off-grid problem. In this paper, atomic norm minimization [20,21,22,23] is introduced for dominant scatterer extraction, to deal with the off-grid issue that the traditional sparse recovery algorithms come with. The scatterers extracted by ANM is verified to be more precise and free of the on-grid constraints, which can improve the performance stability in the low SNR. 

The other problem for ATR based on the HRRP structure features is the aspect sensitivity [2] caused by the change of the aspect angle defined as the angle between the head-on direction of the target and the RLOS. Many existing studies proposed some aspect-frames partition methods [1,24,25,26] to divide the HRRP sequence into the aspect sectors. However, these methods normally require the prior knowledge of target models and omit the aspect angle variations of HRRP sequence in a certain aspect sector, which may be impractical in various environments. Besides, hidden Markov model (HMM) is another widely discussed approach for ATR by HRRP sequence [27,28,29,30,31]. It is realized by training the multi-aspect HRRP sequences using the hidden Markov structure and estimating the state of the test HRRP sequence for recognition. But a large amount of training data is required for possible aspect angles. Recently, in [32], restricted Boltzmann machine was researched for sequential HRRP RATR, which is a supervised learning process and the training process is complicated with many hyper-parameters to tune. In this paper, we introduce the subspace decomposition to improve the robustness to the aspect sensitivity instead. As an effective approach for subspace decomposition, singular value decomposition (SVD) can realize signal denoising by separating the noised data into the signal subspace and the noise subspace [33,34,35,36,37]. The SVD operation projects the HRRP profile matrix onto orthogonal basis spaces decoupled in the range and angle domain [36], in which the range-space singular vectors are referred to constitute the “optimal” features in the range domain [38], while the angle-space singular vectors are not considered in many ATR applications. As the angle domain information is also important in HRRP sequence recognition, in this paper, we exploited the method to combine both the range-space and angle-space singular vectors together for better feature extraction. Moreover, in a short CPI, the migration through resolution cell (MTRC) effect and the high-order Doppler modulation of a target is small among the HRRP sequence, while the background interference more heavily fluctuates with aspect angle variation as it is composed of much weaker backscatterers. Therefore, the SVD process separates the signal subspace from the background interference by abandoning the singular vectors corresponding to smaller singular values, which also helps improve the noise robustness. 

According to the above discussion, a novel SVD-based scatterer matching using atomic norm minimization (SSM-ANM) method is proposed to improve the HRRP-based ATR performance. Firstly, the HRRP sequence is generated with several HRRPs in a CPI with given fluctuating dominant scatterers due to the small change of the aspect angle. Then, the HRRP sequence is further decomposed via SVD, where the left and the right singular vectors correspond to the angle space and the range space, respectively. On one hand, ANM method is utilized to extract the locations and intensities of different dominant scatterers based on the right singular vectors (RSVs), where the Hausdorff distance [19,39] is introduced to get a matching result between the test data and the training data. On the other hand, the angle space correlation is calculated among the test and training data based on the left singular vectors (LSVs). Subsequently, the matching scores for the RSVs and the correlations for the LSVs are fused to form the ATR feature, with the singular values (SVs) used to weigh their corresponding matching results. By the proposed SSM-ANM method, the ATR robustness can be effectively improved in a low SNR scenario. Finally, some results of numerical and outfield experiments are provided to demonstrate the effectiveness of the SSM-ANM method.

The reminder of this paper is organized as follows. In Section 2, the distributed target model is established and the HRRP sequence model is given with relation to the aspect angles. In Section 3, SVD process of the HRRP sequence is firstly discussed. Then, the proposed SSM-ANM method is discussed in detail. In Section 4, some results of numerical experiments and real measured data are provided to demonstrate the effectiveness of the SSM-ANM method. In Section 5, some conclusions are drawn.

Notations: To simplify the presentation, we define the following notations used in this paper. We use bold lower case letters to represent a signal vector, e.g., $a\in C^{N}$, and use bold upper case letters to represent a signal matrix, e.g., $A\in C^{M\times N}$. a∗b is the convolution of a and b. 〈a,b〉 denotes the inner product. The real inner product is defined as 〈a,b〉ℜ=Re(〈a,b〉). ${\left\|a\right\|}_{A}$ is the atomic norm of a under the atom set A. |a| is absolute value of a scaler *a*. pinv(⋅) denotes the pseudo-inverse.

## 2. HRRP Sequence Model for Recognition

**Figure 1:**
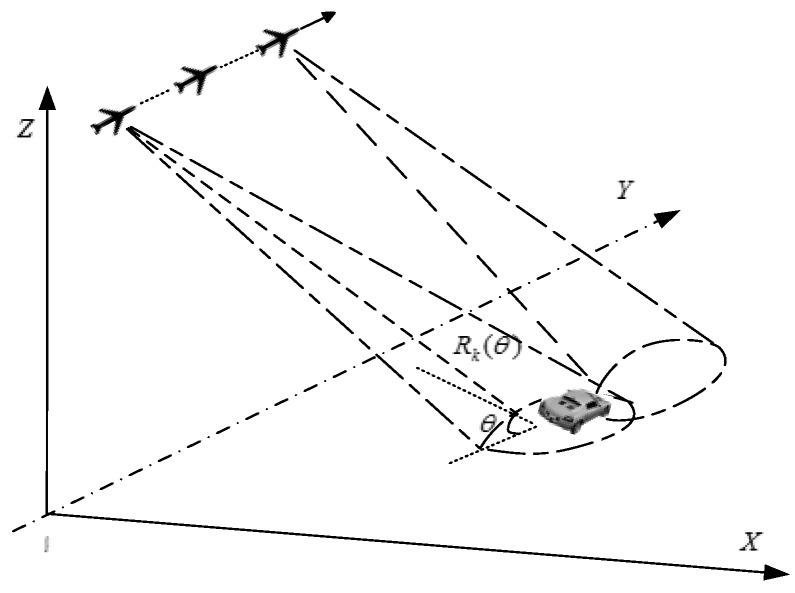
The geometry of the signal model.

Take the airborne radar for ground target recognition as an example. The geometry model is illustrated in Figure 1 for a single-channel airborne wideband radar. It is known that the echoes from large objects at high frequencies can be modeled as a sum of independent scatterers, and the geometric theory of diffraction (GTD) is widely utilized to describe the scatterer model, by which the system frequency response of the target [40] can be written as

(1) $$H\left(f,\theta \right)=\sum_{k=1}^{K}A_{k}\left(\theta \right)\exp\left(j\phi_{k}\left(\theta \right)\right){\left(j\frac{f}{f_{0}}\right)}^{\alpha_{k}}\exp\left[-j\frac{4\pi }{c}R_{k}\left(\theta \right)f\right]$$

where θ is the aspect angle defined as the target-sensor orientation projected on XOY plane, $A_{k}\left(\theta \right)$ and $\phi_{k}\left(\theta \right)$ correspond to the amplitude and phase with given θ. $R_{k}\left(\theta \right)$ represents the radial range of kth scatterer to the radar, *K* denotes the number of scatterers, $f_{0}$ is the center frequency and $\alpha_{k}$ is the type parameter corresponding to the kth scatterers. In applications, the scatterers are supposed to be ideal point scatterers with $\alpha_{k}=0$ [40]. Then the expression of (1) can be further simplified as

(2) $$H\left(f,\theta \right)=\sum_{k=1}^{K}A_{k}\left(\theta \right)\exp\left[j\phi_{k}\left(\theta \right)\right]\exp\left[-j\frac{4\pi }{c}R_{k}\left(\theta \right)f\right]$$

The corresponding response in time domain is denoted by

(3) $$h\left(t,\theta \right)=\sum_{k=1}^{K}A_{k}\left(\theta \right)\exp\left[j\phi_{k}\left(\theta \right)\right]\delta \left(t-\tau_{k}\left(\theta \right)\right)$$

where $\tau_{k}\left(\theta \right)=2R_{k}\left(\theta \right)/c$ indicates the time delay of the kth scatterer’s return.

The transmitted signal of the wideband linear frequency modulated (LFM) waveform can be written as

(4) $$s\left(t\right)=A\mathrm{rect}\left(\frac{t}{T_{p}}\right)\exp\left(2\pi f_{0}t+j\pi \mu t^{2}\right)$$

where *A* is the amplitude of signal, $T_{p}$ is the pulse width, and rect(⋅) is the rectangular function. $\mu =2B/T_{p}$ corresponds to the chirp rate, in which *B* is the bandwidth. The amplitude spectrum of s(t) is represented by

(5) $$\left|S\left(f\right)\right|=\{\begin{array}{ll} A\sqrt{\frac{T_{p}}{B}}, & \left|f-f_{0}\right|\leq B/2\\ 0, & \left|f-f_{0}\right|>B/2 \end{array}$$

The received signal $s_{r}\left(t,\theta \right)$ can be regarded as the convolution of the transmitted signal s(t) and the target system response h(t,θ), i.e.,

(6) $$s_{r}\left(t,\theta \right)=s\left(t\right)\times h\left(t,\theta \right)$$

After matched filtering the output signal $s_{o}\left(t,\theta \right)$ can be represented as

(7) $$s_{o}\left(t,\theta \right)=s\left(t\right)\times h\left(t,\theta \right)\times s\left(-t\right)$$

in which the transmitted waveform is also chosen as the matched filter. Thus, its spectrum $S_{o}\left(f,\theta \right)$ is derived as

(8) $$\begin{array}{l} S_{o}\left(f,\theta \right)={\left|S\left(f\right)\right|}^{2}H\left(f,\theta \right)\\ \;=\frac{A^{2}T_{p}}{B}\sum_{k=1}^{K}A_{k}\left(\theta \right)\exp\left[j\phi_{k}\left(\theta \right)\right]\exp\left[-j\frac{4\pi }{c}R_{k}\left(\theta \right)f\right],\;\left|f-f_{0}\right|<B/2 \end{array}$$

By the inverse Fourier transform of $S_{o}\left(f,\theta \right)$, the target echo $s_{o}\left(t,\theta \right)$ is further expressed as

(9) $$\begin{array}{ll} s_{o}\left(t,\theta \right) & =\int_{w}{\left|S\left(f\right)\right|}^{2}H\left(f,\theta \right)\exp\left(2\pi ft\right)df\\ & =C_{0}\sum_{k=1}^{K}A_{k}\left(\theta \right)\exp\left[j\phi_{k}\left(\theta \right)\right]\mathrm{sinc}\left[\pi B\left(t-\tau_{k}\left(\theta \right)\right)\right]\exp\left[j2\pi f_{0}\left(t-\tau_{k}\left(\theta \right)\right)\right] \end{array}$$

where $C_{0}=A^{2}T_{P}$. According to (9), as the combination of echoes of different scatterers, the HRRPs vary versus the aspect angle θ. During the CPI, several pulses of echoes are received and constitute the HRRP sequence, where the discrete form and frequency response after digital down-conversion can be written as

(10) $$s_{o}\left(n,m\right)=C_{0}\sum_{k=1}^{K}A_{k}\left(\theta_{n}\right)\exp\left[j\phi_{k}\left(\theta_{n}\right)\right]\mathrm{sinc}\left[\pi B\left(m\Delta t-\tau_{k}\left(\theta_{n}\right)\right)\right]\exp\left(-j2\pi f_{0}\tau_{k}\left(\theta_{n}\right)\right)$$

(11) $$z_{o}\left(n,l\right)=\frac{C_{0}}{B}\sum_{k=1}^{K}A_{k}\left(\theta_{n}\right)\exp\left[-j\frac{4\pi }{c}R_{k}\left(\theta_{n}\right)l\Delta f+j\phi_{k}\left(\theta_{n}\right)\right]$$

in which m,l represent the *m*th time sampling and *l*th frequency response respectively, where m∈{1,⋯,M} and l∈{1,⋯,L}. Δt and Δf denote the time and frequency interval respectively. n represents the pulse number in the CPI, and $\theta_{n}$ is the aspect angle corresponding to the *n*th pulse.

To combine the HRRP sequence in the CPI for recognition, the MTRC caused by the relative motion between target and radar needs to be compensated first. For the ground target whose motion can be omitted in the CPI as it is far smaller than the motion of the airborne radar, the MTRC is compensated directly by the velocity information of the cooperative airborne platform. For the air target, the techniques for range alignment and phase compensation are widely researched [41,42], which will not be discussed here. After motion compensation, fluctuations still remain for the scatterers caused by the aspect angle variation, which can be appropriately applied for recognition in our following discussion in Section 3. 

## 3. The Proposed HRRP Sequence Matching Method

In this part, a novel method of SVD-based scatterer matching using atomic norm minimization (SSM-ANM) is demonstrated for feature extraction and recognition. Firstly, SVD process for the HRRP sequence is explored in Section 3.1, verifying the effectiveness in noise reduction. Secondly, the whole process for SSM-ANM is illuminated in detail in Section 3.2. Then the implementation of the key procedures in SSM-ANM is introduced in Section 3.3. Lastly, some remarks on the proposed SSM-ANM method are concluded.

### 3.1. Singular Value Decomposition of the HRRP Sequence

Assume that the MTRC effect caused by the relative motion between the platform and target has been compensated as discussed in Section 2. Moreover, the HRRP part corresponding to the target needs to be determined in advance [43]. Then, the received HRRP sequence of the target area in the CPI can be denoted by

**Figure 2:**
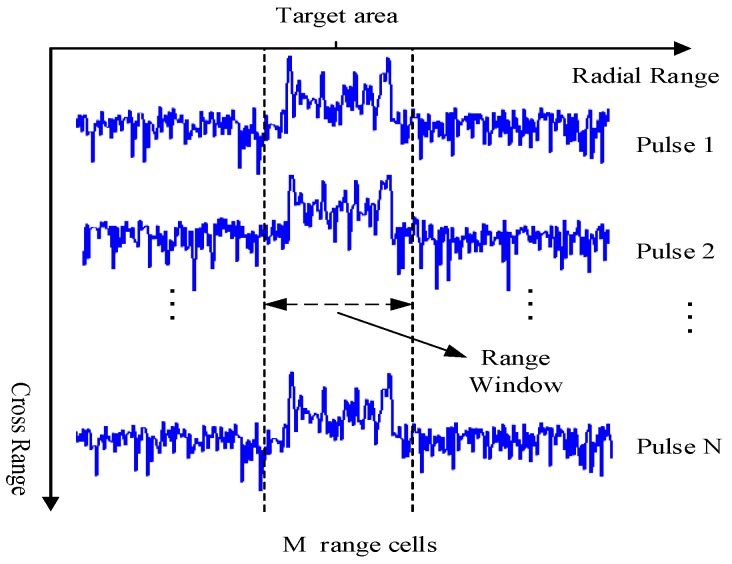
The received HRRPs in the CPI after MTRC compensation.

(12) X=S+N

where $X={\left[x\right]}_{n,m}$, $S={\left[s\right]}_{n,m}$ and $N={\left[n\right]}_{n,m}$, which represent the received signal, target echoes, and background noise respectively in each pulse index *n* and range cell index *m*, where 1≤m≤M, 1≤n≤N, as shown in Figure 2. *N* is the pulse number of a CPI and *M* is the range cell number of the target area, normally M>N. 

The singular value decomposition (SVD) of X is given by

(13) $$X=U\Lambda V^{T}$$

where the columns of $U\in {\text{ℂ}}^{N\times N}$ and $V\in {\text{ℂ}}^{M\times M}$ correspond to the LSVs and RSVs of X respectively. $\Lambda \in {\text{ℝ}}^{N\times M}$ is a diagonal matrix containing the *N* SVs of X in descending order that $\lambda_{1}\geq \lambda_{2}\geq \ldots \geq \lambda_{N}\geq 0$. Assume that the background noise in each range cell obeys a complex Gaussian distribution with the mean of 0 and variance of $\sigma_{n}^{2}$ with independent identically distribution (i.i.d).

Next, consider the singular vectors as follows. According to (13), the RSVs in **V** span the orthogonal basis space in the range domain, while the LSVs in **U** span the basis space in the angle domain [38]. Given the Eckhart and Young theorem [44], the top singular vectors with the largest SVs can well approximate the signal, called the signal subspace, while the tail vectors with smallest SVs span the noise subspace. By reconstructing the signal with the top singular vectors, background interference can be reduced as it corresponds to the tail singular vectors. The energy threshold is set as η, defined by $\eta =\sum_{i=1}^{Q_{S}}\lambda_{i}/\sum_{i=1}^{N}\lambda_{i}$. Thus $Q_{S}$, the number of SVs to be chosen, can be determined by η. 

In the work of the state-of-the-art for recognition, the RSVs in **V** are utilized for ATR while the LSVs are ignored [36,37,38]. However, the information in **U** is also useful in our HRRP sequence based recognition. Thus, we exploit the SVD-based feature extraction method by jointly using RSVs and LSVs, called the SSM-ANM method which will be discussed in detail in the following part. 

### 3.2. The Process of the SSM-ANM Method for Recognition 

The training data is denoted by ${\left\{X_{N\times M_{d,g}}\right\}}^{d,g}$, where $X_{N\times M_{d,g}}$ is the target HRRP sequence in a CPI, in which *N* is the pulse number and $M_{d,g}$ is the range cell number. d∈{1,⋯,D} and g∈{1,⋯,G} represent the posture number and the type number of the targets respectively. The test data is denoted by $Y_{N\times M}$, and the recognition task aims to find its right type.

The LSVs, RSVs and SVs for the training data are represented by ${\left\{u_{1},u_{2},\cdots ,u_{N}\right\}}^{d,g},\;{\left\{v_{1},v_{2},\cdots ,v_{M_{d,g}}\right\}}^{d,g}$ and ${\left\{\lambda_{1},\lambda_{2},\cdots ,\lambda_{N}\right\}}^{d,g}$, while the LSVs, RSVs and SVs for the test data are $\left\{u_{1}^{0},u_{2}^{0},\cdots ,u_{N}^{0}\right\},\;\left\{v_{1}^{0},v_{2}^{0},\cdots ,v_{M}^{0}\right\}$ and $\left\{\lambda_{1}^{0},\lambda_{2}^{0},\cdots ,\lambda_{N}^{0}\right\}$. As the signal can be well approximated by the singular vectors corresponding to the largest SVs, here we use s,t to represent the number of the dominant SVs for the training and test data, respectively, which can be determined by the energy threshold η. 

For all target types g=1,⋯,G and postures d=1,⋯,D, loop and calculate the corresponding matching scores. The type $g_{0}$ with the largest score is the recognition result. The whole algorithmic recap for the ground target recognition based on SSM-ANM algorithm is demonstrated in Table 1. 

In the SSM-ANM procedures above, for the training data $X_{N\times M_{d,g}}^{d.g}$ and test data $Y_{N\times M}$, their range space matching matrix is denoted by $M_{s\times t}^{d,g}$, while their angle space correlation matrix is $Z_{s\times t}^{d.g}$. $\Lambda_{s\times s}^{d,g}$ and $\Lambda_{t\times t}$ are diagonal matrices consisted by their dominant SVs respectively, which are pre-normalized by their traces. Then, the matching score $o^{d.g}$ is fused by the range space matching result and the angle space correlation as follows.

**Figure 3:**
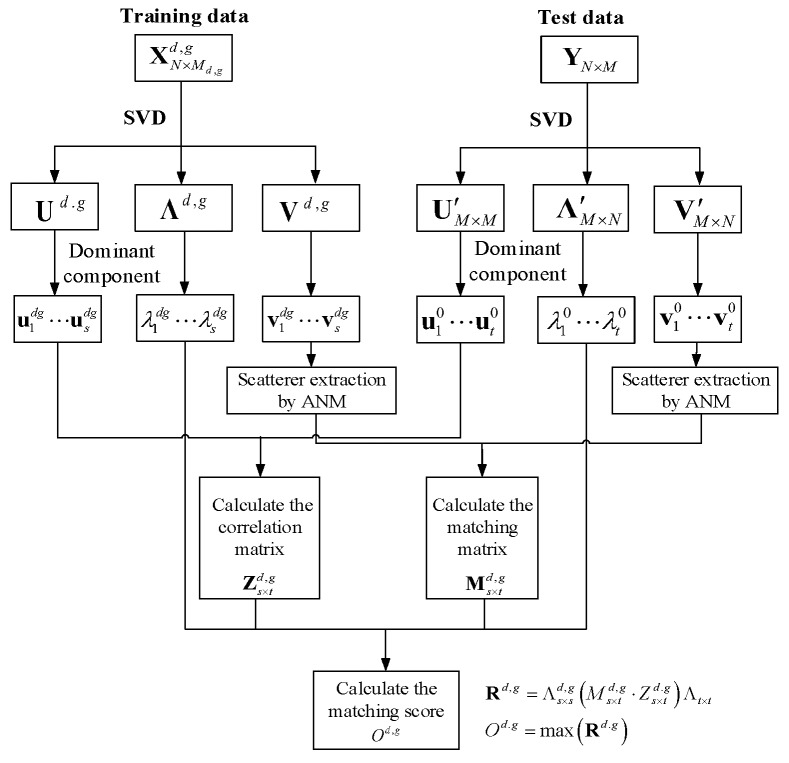
Implementation flowchart of the SSM-ANM method.

The matching matrix is defined by

(14) $$R^{d,g}=\Lambda_{s\times s}^{d,g}\left(M_{s\times t}^{d,g}\cdot Z_{s\times t}^{d.g}\right)\Lambda_{t\times t}$$

where (⋅) is the dot product for the matrix. Equation (14) can be seen as the fusion result of $M_{s\times t}^{d,g}$,$Z_{s\times t}^{d.g}$, $\Lambda_{s\times s}^{d,g}$ and $\Lambda_{t\times t}$. Furthermore, the matching score $o^{d.g}$ is represented by

(15) $$o^{d.g}=\max\left(R^{d.g}\right)$$

where max(⋅) is the operation to get the largest entry of the matrix. $o^{d.g}$ is the output matching score with given type *g* and posture *d*. The flowchart of the SSM-ANM is illustrated in Figure 3.

### 3.3. Implemantation of the Key Procedures in SSM-ANM

In this section, the key procedures in SSM-ANM are illustrated in detail, including the scatterer extraction by ANM, definition of the range space matching matrix $M_{s\times t}^{d,g}$ and the angle correlation matrix $Z_{s\times t}^{d.g}$, which corresponding to Section 3.3.1, Section 3.3.2 and Section 3.3.3, respectively.

#### 3.3.1. Dominant Scatterer Extraction by Atomic Norm Minimization

In this part, the dominant scatterer extraction by ANM is researched. In our former work [23], the dominant scatterers are extracted by single HRRP, which may be unsteady in lower SNR. As the dominant RSVs of the HRRP sequence contain the scatterer information in the range domain with less noise interference, here we utilize ANM to extract the scatterer features based on dominant RSVs instead of the single HRRP. Moreover, it will be demonstrated in Section 4 that the larger RSVs follows the scatterer model which contains the information for more steady scatterers, and the performance of the dominant RSVs is superior to single HRRP in scatterer extraction and recognition. According to [23], the frequency response for scatterers can be denoted by

(16) $$z_{1}\left(l\right)=\sum_{k=1}^{K}A_{k}\exp\left[-j2\pi \frac{2R_{k}}{c}\Delta fl+j\phi_{k}\right],\;l=0,\ldots ,L-1$$

which indicates that the signal in frequency domain can be modeled as the sum of *K* sinusoid components corresponding to the *K* dominant scatterers. As the number *K* is usually far smaller than the sampling frequency numbers, it can be solved by the ANM [20].

Atoms of ANM are constructed by a(f,φ)∈C, f∈[0,1] and φ∈[0,2π) as

(17) $${\left[a\left(f,\varphi \right)\right]}_{l}=\exp\left[j\left(2\pi fl+\varphi \right)\right]$$

Then (16) can be represented by the atoms as

(18) $$z_{1}\left(l\right)=\sum_{k=1}^{K}A_{k}{\left[a\left(f_{k},\phi_{k}\right)\right]}_{l}$$

where

(19) $$f_{k}=-2R_{k}\Delta f/c$$

The set of atoms is denoted by A={a(f,ϕ):f∈[0,1],ϕ∈[0,2π)}, and the atomic norm ${\left\|\cdot \right\|}_{A}$ represents its unit ball with the convex hull of A:

(20) $$\begin{array}{ll} {\left\|x\right\|}_{A} & =\;\inf\left\{t>0:x\in t\mathrm{conv}\left(A\right)\right\}\\ & =\underset{\begin{array}{c} c_{k}\geq 0\\ \phi_{k}\in \left[0,2\pi \right)\\ f_{k}\in \left[0,1\right] \end{array}}{\inf}\left\{\sum_{k}c_{k}:x=\sum_{k}c_{k}a\left(f_{k},\phi_{k}\right)\right\}. \end{array}$$

The atomic norm minimization problem is

(21) $$\begin{array}{l} \mathrm{minimize}{\left\|x\right\|}_{A}\\ \mathrm{subject}\;\mathrm{to}\;x_{j}=x_{j}^{\#},j\in T \end{array}$$

where *T* represents the subset of entries we observe and $x_{j}^{\#}$ is the continuous signal. The frequency of $f_{k}$ can be localized by the dual optimal solution [20] introduced as follows. Define the inner product as $\langle q,x\rangle =x^{*}q$, and the real inner product as 〈q,x〉ℜ=Re(x∗q). Next, the dual norm of ${\left\|\cdot \right\|}_{A}$ is defined as

(22) $$\begin{array}{ll} {\left\|q\right\|}_{A}^{*} & =\underset{{\left\|x\right\|}_{A}\leq 1}{\sup}{\langle q,x\rangle }_{ℜ}\\ & =\underset{\phi \in \left[0,2\pi \right),f\in \left[0,1\right]}{\sup}{\langle q,e^{i\phi }a\left(f,0\right)\rangle }_{ℜ}\\ & =\underset{f\in \left[0,1\right]}{\sup}\left|\langle q,a\left(f,0\right)\rangle \right| \end{array}$$

According to [20], the dual atomic norm is equal to the maximum modulus of the polynomial $Q\left(z\right)=\sum_{j\in J}q_{j}z^{-j}$ on the unit circle. The dual problem of (21) can be solved by

(23) $$\begin{array}{ll} \underset{q}{\mathrm{maximize}} & {\langle q_{T},x_{T}^{\#}\rangle }_{R}\\ \mathrm{subject}\;\mathrm{to} & {\left\|q\right\|}_{A}^{*}\leq 1\\ & q_{T^{c}}=0 \end{array}$$

Therefore, the estimation ${\hat{f}}_{k}$ of frequency $f_{k}$ can be determined by $Q\left(f\right)=\langle q,a\left(f,0\right)\rangle :=\sum_{j\in J}q_{j}e^{-i2\pi jf}$, which satisfies

(24) $$\begin{array}{l} Q\left(f_{k}\right)=\mathrm{sign}\left(c_{k}\right),\forall f_{k}\in \Omega \\ \left|Q\left(f\right)\right|<1\;\forall f\notin \Omega \\ q_{j}=0\;\forall j\notin T \end{array}$$

With ${\hat{f}}_{k}$, the scatterer location estimation ${\hat{R}}_{k}$ can be calculated by (19). The corresponding complex amplitude ${A^{\prime }}_{k}$ can be solved from (16) by

(25) $$\left[\begin{array}{cccc} \exp\left(-j2\pi {\hat{f}}_{1}\right) & \exp\left(-j2\pi {\hat{f}}_{2}\right) & \cdots & \exp\left(-j2\pi {\hat{f}}_{K_{1}}\right)\\ \exp\left(-j2\pi \cdot 2{\hat{f}}_{1}\right) & \exp\left(-j2\pi \cdot 2{\hat{f}}_{2}\right) & \cdots & \exp\left(-j2\pi \cdot 2{\hat{f}}_{K_{1}}\right)\\ \vdots & \vdots & \ddots & \vdots \\ \exp\left(-j2\pi \cdot L{\hat{f}}_{1}\right) & \exp\left(-j2\pi \cdot L{\hat{f}}_{2}\right) & \cdots & \exp\left(-j2\pi \cdot L{\hat{f}}_{K_{1}}\right) \end{array}\right]\left[\begin{array}{c} {A^{\prime }}_{1}\\ {A^{\prime }}_{2}\\ \vdots \\ {A^{\prime }}_{K_{1}} \end{array}\right]=\left[\begin{array}{c} z_{1}\left(1\right)\\ z_{1}\left(2\right)\\ \vdots \\ z_{1}\left(L\right) \end{array}\right]$$

which equals to

(26) $$A^{\prime }=\mathrm{pinv}(\Phi )z$$

where

(27) $$A^{\prime }=\left[\begin{array}{c} {A^{\prime }}_{1}\\ {A^{\prime }}_{2}\\ \vdots \\ {A^{\prime }}_{K_{1}} \end{array}\right],\;z=\left[\begin{array}{c} z_{0}\left(1\right)\\ z_{0}\left(2\right)\\ \vdots \\ z_{0}\left(L\right) \end{array}\right],\;\Phi =\left[\begin{array}{cccc} \exp\left(-j2\pi {\hat{f}}_{1}\right) & \exp\left(-j2\pi {\hat{f}}_{2}\right) & \cdots & \exp\left(-j2\pi {\hat{f}}_{K_{1}}\right)\\ \exp\left(-j2\pi \cdot 2{\hat{f}}_{1}\right) & \exp\left(-j2\pi \cdot 2{\hat{f}}_{2}\right) & \cdots & \exp\left(-j2\pi \cdot 2{\hat{f}}_{K_{1}}\right)\\ \vdots & \vdots & \ddots & \vdots \\ \exp\left(-j2\pi \cdot L{\hat{f}}_{1}\right) & \exp\left(-j2\pi \cdot L{\hat{f}}_{2}\right) & \cdots & \exp\left(-j2\pi \cdot L{\hat{f}}_{K_{1}}\right) \end{array}\right]$$

where pinv(⋅) denotes the pseudo-inverse, and $K_{1}$ represents the scatterer number, which may be larger or smaller than the real scatterer number *K*. The amplitude and phase estimation ${\hat{A}}_{k}$, ${\hat{\phi }}_{k}$ can be obtained from ${A^{\prime }}_{k}$.

The output of ANM dominant scatterer extraction is a set denoted by the location and intensity of each scatterer $P_{k}$, expressed by the feature set as

(28) $$\mathbb{P}=\left\{P_{1},P_{2},\cdots ,P_{K_{1}}\right\}=\left\{\left({\hat{R}}_{1},{\hat{A}}_{1}\right),\left({\hat{R}}_{2},{\hat{A}}_{2}\right),\cdots ,\left({\hat{R}}_{K_{1}},{\hat{A}}_{K_{1}}\right)\right\}$$

Compared to the scatterer extraction by OMP, ANM is a sparse recovery method without a prebuild discrete dictionary, which can get rid of the off-grid problem. Thus, the estimation precision can be increased by ANM, which will be verified in Section 4.1.

The output scatterer set ℙ will be used to compute the dominant-scatterers’ Hausdorff distance [19] between the training data and test data, which is introduced in detail in the Section 3.3.2.

#### 3.3.2. Scatterer Matching for the Dominant RSVs by Dominant-Scatterers’ Hausdorff Distance

According to Section 3.3.2, the scatterer feature set of the test target extracted by ANM based on the sum of *K* sinusoid components model is denoted by $\left\{{\text{ℙ}}^{0}\right\}$. Similarly, the scatterer feature sets of the training data are denoted by ${\left\{\text{ℙ}\right\}}^{d,g}$ for *g* = 1, …, *G* and *d* = 1, …, *D*. As the Hausdorff measure is widely used for calculating the distance between two point sets, here, we utilize the Hausdorff distance proposed in [19], called dominant-scatterers’ Hausdorff distance(ds-HD) for the scatterer matching of the dominant RSVs. Given two point sets $A=\{a_{1},a_{2},\cdots ,a_{N}\}$ and $B=\{b_{1},b_{2},\cdots ,b_{Q}\}$, the ds-HD is expressed as

(29) $$\begin{array}{l} h_{ds}\left(A,B\right)=\frac{1}{P_{1}}\sum_{a_{n_{1}}\in A^{\prime }}\underset{b_{q_{1}}\in B^{\prime }}{\min\;}d\left(a_{n_{1}},b_{q_{1}}\right)\\ h_{ds}\left(B,A\right)=\frac{1}{P_{2}}\sum_{b_{q_{1\in B^{\prime }}}}\underset{a_{n_{1}}\in A^{\prime }}{\min\;}d\left(b_{q_{1}},a_{n_{1}}\right) \end{array}$$

where $A^{\prime }=A_{1}^{\prime }\cup \{a_{P_{1}}\}=\{a_{1},a_{2},\cdots ,a_{P_{1}-1}\}\cup \{a_{P_{1}}\}$, $A^{\prime }\subset A$, $P_{1}<N$, and $B^{\prime }=B_{1}^{\prime }\cup \{b_{P_{2}}\}=\{b_{1},b_{2},\cdots ,b_{P_{2}-1}\}\cup \{b_{P_{2}}\}$, $B^{\prime }\subset B$, $P_{2}<Q$. Let A and B be the feature sets corresponding to one dominant RSV of the training data and the test data respectively. The d(⋅,⋅) is calculated from the Mahalanobis distance

(30) $$d\left(a_{n_{1}},b_{q_{1}}\right)=\sqrt{{\left(a_{n_{1}}-b_{q_{1}}\right)}^{T}\Sigma^{-1}\left(a_{n_{1}}-b_{q_{1}}\right)}$$

where Σ is a diagonal matrix with diagonal entries consisted by the measurement error variance for each feature of the scatterers, which can be estimated by the training data. Then, the ds-HD represents the mean distance from one point set to another. According to (28), two features extracted by ANM are chosen, one is the location index of the scatterer and the other is its intensity. Finally, the ds-HD used here is utilized to evaluate the distance between the training and test data as

(31) $$H(A,B)=\max\left(h_{ds}\left(A,B\right),h_{ds}\left(B,A\right)\right)$$

To convert the ds-HD to the matching result of the two-point set, we introduce the matching degree index(MDI) MDI(A,B) as the reciprocal of H(A,B) as

(32) MDI(A,B)=1/H(A,B)

Then, the MDI for the dominant RSVs of $\left\{v_{1}^{0},\cdots ,v_{t}^{0}\right\}$ and $\left\{v_{1}^{d,g},\cdots ,v_{s}^{d,g}\right\}$ can be calculated according to the above calculation (29)–(32), denoted by the range profile matching matrix $M={\left[m_{i,j}\right]}_{s\times t}$, where $m_{i,j}$ represents the MDI of the vector $v_{i}^{0}$ and $v_{j}^{d,g}$.

#### 3.3.3. Angle Space Correlation by the LSVs

**Figure 4:**
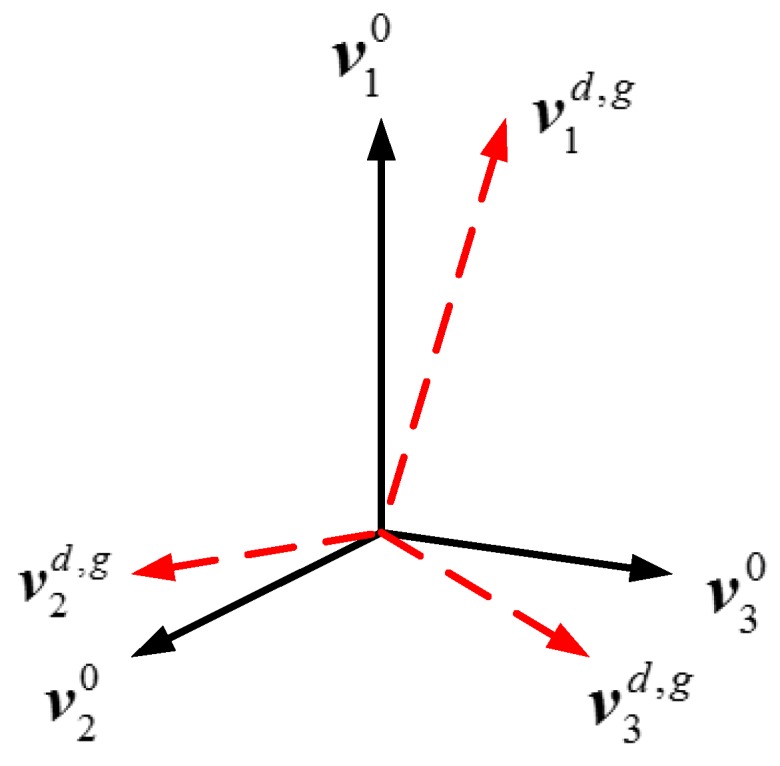
The angle between the spaces that the training and test data spans.

Moreover, the basis space for each RSV can be spanned in the angle domain by the corresponding LSV. Therefore, the dominant LSVs are exploited to obtain the angle space correlation between the training and test data. For better demonstration, take s=t=3 as example. As illustrated in Figure 4, the angle space $\left\{v_{1}^{0},v_{2}^{0},v_{3}^{0}\right\}$ that the $\left\{u_{1}^{0},u_{2}^{0},u_{3}^{0}\right\}$ spans do not coincide with the space $\left\{v_{1}^{d,g},v_{2}^{d,g},v_{3}^{d,g}\right\}$ that $\left\{u_{1}^{d,g},u_{2}^{d,g},u_{3}^{d,g}\right\}$ spans. The larger angle between them, the bigger difference their signal spaces have, which should be included in the recognition process. Therefore, we define the angle space correlation matrix as

(33) $$\begin{array}{l} Z={\left[z_{i,j}\right]}_{s\times t}\\ z_{i,j}=\mathrm{corr}(u_{i}^{0},u_{j}^{d,g}) \end{array}$$

where corr(·) is the correlation operation. Considering that $v_{1}^{0},v_{2}^{0},v_{3}^{0}$ have different energy according to their SVs $\lambda_{1}^{0},\lambda_{2}^{0},\lambda_{3}^{0}$, they should be given with different weight to decide the recognition result, as used in [38]. Similarly, the weight for $v_{1}^{d,g},v_{2}^{d,g},v_{3}^{d,g}$ should be determined according to $\lambda_{1}^{d,g},\lambda_{2}^{d,g},\lambda_{3}^{d,g}$.

### 3.4. Some Remarks on the Proposed SSM-ANM Method

**Remark 1.**  In the SSM-ANM method, the number of the dominant singular vectors for the training data can be different from that for the test data, which is more flexible than the traditional pattern recognition methods. Moreover, the number is determined according to the environment noise and the aspect angle variation during the CPI. With higher SNR and smaller aspect angle variation, the fewer number of singular vectors contains the signal subspace, making the SSM-ANM method adaptive to various cases.

**Remark 2.**  In the Section 3.3.1, we firstly extract the scatterers on dominant RSVs by ANM and then calculate the ds-HD between the training and test data. Compared with the template matching method which calculates the RSVs’ correlation between the training and test data directly, the scatterer extraction procedure abandons the noise-only range cells and, thus, improves the performance stability in lower SNR without information loss as the structure information is mostly determined by the dominant scatterers.

**Remark 3.**  The paper mainly focuses on the feature extraction stage of ATR, thus, only the scatterer matching by ds-HD is used as the classifier to compare the performances of the SSM-ANM method with the state-of-the-art literature in feature extraction. In our future work, more classifiers will be tested such as support vector machine (SVM), random forest (RF) or others for possible better classification performance.

## 4. Numerical and Outfield Experiments

In this section, the results of both numerical simulations and outfield experiments are given to evaluate the performance of the proposed SSM-ANM recognition accordance with different scenarios.

### 4.1. Effectiveness of ANM in Dominant Scatterer Extraction

To validate the effectiveness of the SVD-based dominant scatterer extraction using ANM, a simple scenario is designed as Figure 1. The airborne radar is flying towards the target with radial range $R_{0}=10$ km and aspect angle θ= 30°, in which the target is consisted by six dominant scatterers from **A** to **F**. The relative radial range of the six scatterers as well as their intensities are listed in Table 2, where **C** and **D** have the largest and stable intensity and the other smaller ones have fluctuate intensity following Rayleigh distribution. The signal is modeled as (8)–(11), and the system parameters are listed as Table 3. 

**Figure 5:**
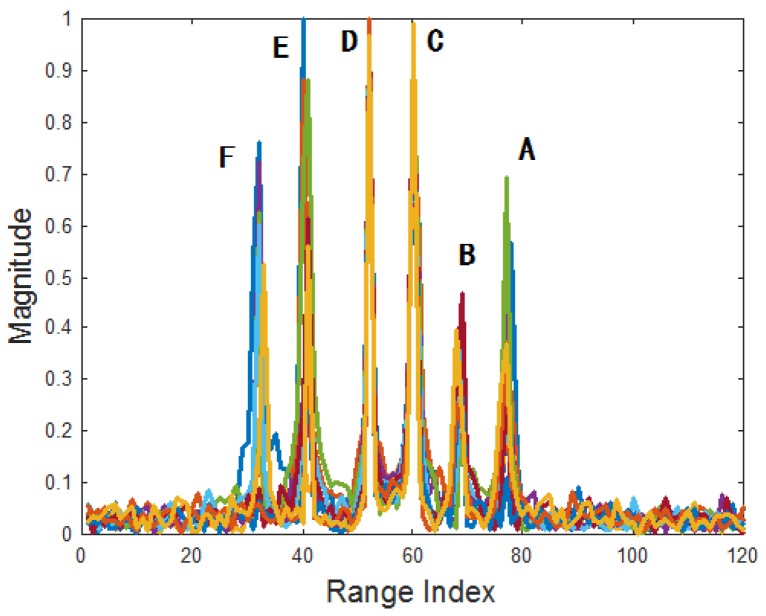
The HRRP sequence of the given target.

**Figure 6:**
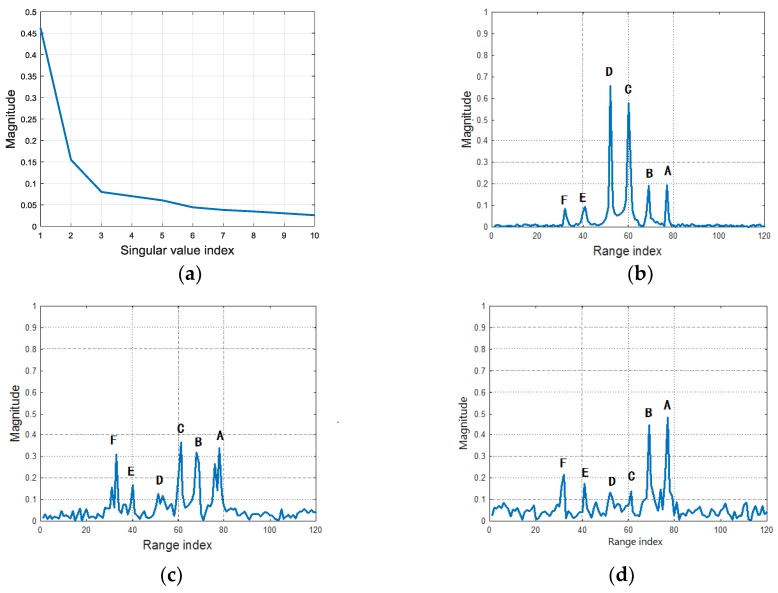
SVs and dominant RSVs of the HRRP sequence. (**a**) Singular values of HRRPs; (**b**) RSV corresponding to the 1st singular value; (**c**) RSV corresponding to the 2nd singular value; (**d**) RSV corresponding to the 3rd singular value.

During the flight, the aspect angle varies with the relative positions between target and radar. HRRPs are synthesized and the MTRC between them is compensated with SNR = 30 dB. Figure 5 shows the HRRP sequence in the CPI, where the six dominant scatterers are noted. As the scatterers **A**, **B**, **E** and **F** have fluctuate intensity, their profiles vary seriously. After SVD, the SVs are plotted in Figure 6a, where most energy falls on the first three largest ones, and the smaller SVs only contain the noise. Figure 6b–d give the RSVs corresponding to the 1st, 2nd, 3rd largest SVs. It can be seen that the 1st RSV is consisted by the most stable components of the HRRP sequence, thus the scatterer **C** and **D** have more energy in it. The 2nd singular value contains more fluctuate components where scatterer **F**, **E**, **B** and **A** are more prominent than **C** and **D**. As to the 3rd singular value, **C** and **D** are even weaker than the others, nearly submerged by the noise. 

**Figure 7:**
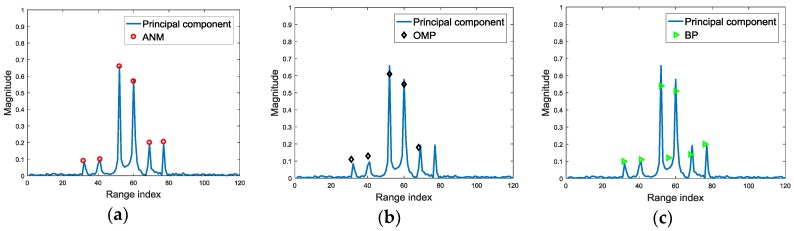
The scatterer extraction comparison for ANM, OMP and BP. (**a**) The scatterer extraction result via ANM; (**b**) The scatterer extraction result via OMP; (**c**) The scatterer extraction result via BP.

The scatterer extraction results on the largest RSV by ANM, OMP and BP are illustrated in Figure 7, where ANM is more accurate on the estimations of scatterer location and intensity than OMP and BP. 

The simulations above verify that, after SVD, the noise is reduced in the dominant RSVs, which increases the accuracy of scatterer extraction. Moreover, the most stable components of HRRPs are focused on the RSVs corresponding to the largest SVs. Moreover, ANM shows superiority in dominant scatterer extraction than OMP and BP, which is free of the off-grid problem.

### 4.2. Performance of the SSM-ANM Method for Recognition

#### 4.2.1. The Effectiveness of the LSVs for Target Recognition

In the following discussion, we evaluate the effectiveness of the LSVs in feature extraction and recognition. For the comparison convenience, we define the matching matrix and matching score calculated without the angle space correlation matrix by the LSVs as

(34) $$R_{r}^{d,g}=\Lambda_{s\times s}^{d,g}M_{s\times t}^{d,g}\Lambda_{t\times t}$$

(35) $$O_{r}^{d,g}=\max(R_{r}^{d,g})$$

HRRP sequences are generated corresponding to four types of military vehicles with SNR = 20 dB. The professional software 3DS MAX and FEKO are used to generate the scatterer model as well as the echoes of four types of military vehicles. Furthermore, the system parameters of the following simulations are listed in Table 3, which also act as the system parameters for the outfield experiments in Section 4.2.3. Then, the training HRRP sequences for the four types are synthesized in aspect angle centers 0, 1°, 2°, …, 180°, with angle variation of 1° in each CPI. Thus, we have 181 training samples for each target. The test HRRP sequences are generated with random aspect angle centers in 0–180°, with angle variation of 0.5°, 1° and 2°. 

Two hundred Monte Carlo simulations are carried out to calculate the matching score between the training and test HRRP sequences as defined in (15) and (35). In Table 4, the average maximum matching score in each simulation for arbitrary two types are listed, where the matching score (35) is compared with the matching score (15) in bold font. The results indicate that by utilizing LSVs, the matching scores among different types are decreased, which effectively improves the robustness to the aspect-sensitivity in a degree. 

#### 4.2.2. Recognition Performance Comparisons 

**Figure 8:**
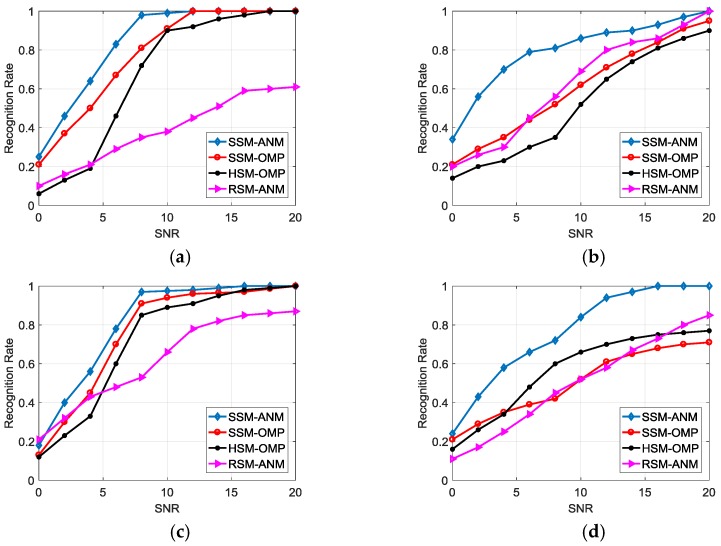
Recognition performance comparison in different SNRs. (**a**) Type A; (**b**) Type B; (**c**) Type C; (**d**) Type D.

The recognition performance for the proposed SSM-ANM method is compared with the SVD-based scatterer matching by OMP (SSM-OMP), the HRRP-based scatterer matching by OMP (HSM-OMP) proposed in [18], as well as the method using the matching score $O_{r}^{d,g}$ in (35) without LSVs (denoted by RSM-ANM). We generate echoes of the four targets in the same way discussed in Section 4.2.1, with 200 times of Monte Carlo simulations carried out. We investigate the recognition performance in different SNRs. Figure 8 compares the recognition rates of the four types of targets for the four methods, with test data varying from 0 to 20 dB and training data of 20 dB. The result indicates the advantage of the SSM-ANM in HRRP-based feature extraction and recognition.

**Figure 9:**
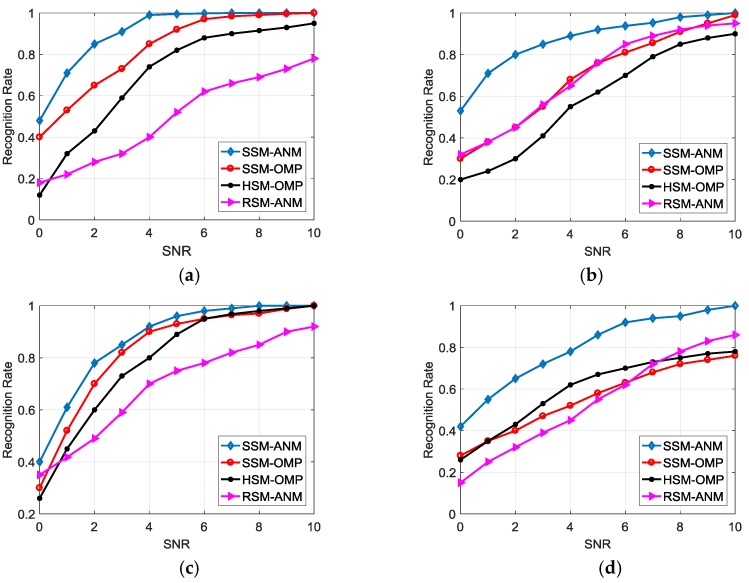
Recognition performance comparison in different SNRs (CPI = 20). (**a**) Type A; (**b**) Type B; (**c**) Type C; (**d**) Type D.

Next, we consider the performances of the four methods in extended CPI, where Figure 9 demonstrates the recognition results with CPI = 20. The SSM-ANM method still has good performances, as it can adaptively determine the number of dominant SVs when the CPI extends to more pulses and energy spreads to more SVs. But the computation complexity will increase and the data rate will decrease for recognition when the CPI is too large. In our system, CPI in the range of 10–20 pulses is a better choice for the ATR task.

The computational complexity of the four methods is listed in Table 5. Although the SSM-ANM method has the largest computational complexity, some fast approaches for SVD and ANM are researched to reduce the complexity further [45,46,47].

#### 4.2.3. Outfield Real Data Experiment

**Figure 10:**
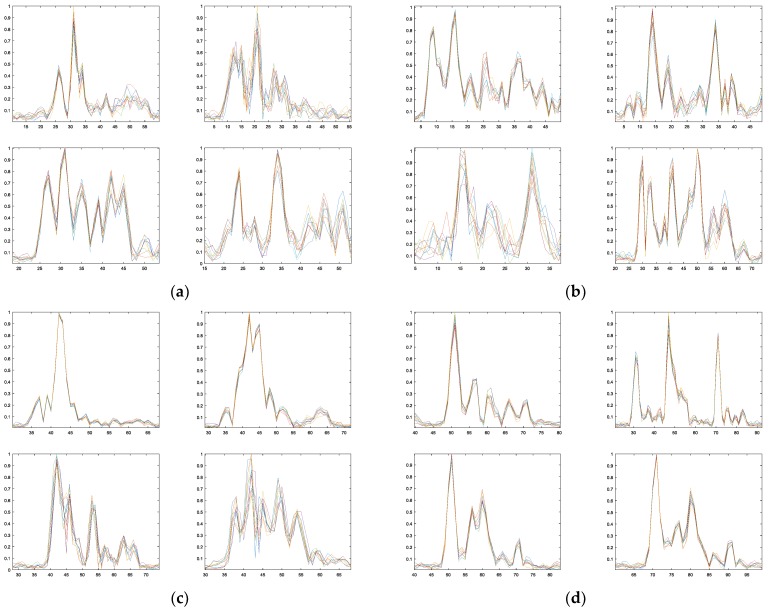
Typical HRRP sequences for the four types of vehicles (CPI = 10). (**a**) Type A; (**b**) Type B; (**c**) Type C; (**d**) Type D.

Outfield real data experiments are carried out with the same parameters in Table 3. The echoes for the four typical vehicles in different aspect angles are processed and their HRRPs are generated for recognition, as illustrated in Figure 10. CPI is set as ten pulses and 500 HRRP sequences in each aspect angle center are obtained, with five angle centers from 0° to 180° with step of 45° and about 1° angle variation. Every fifth HRRP sequence is used for training and the rest are used for testing. Thus, we have 100 sequences for training and 400 sequences for testing in each aspect angle center. The SNR for the training and test data is about 30 dB. The radical range is about 5 km. The recognition results are listed in Table 6, Table 7, Table 8 and Table 9, which verified the effectiveness of the SSM-ANM method in scatterer matching and recognition.

## 5. Conclusions

In this paper, we proposed a scatterer matching method of SSM-ANM by HRRP sequence to get the dominant scatterer features. Firstly, the HRRP sequence in a CPI is first decomposed by SVD. Then, the dominant RSVs are processed by ANM to extract the dominant scatterer information, while the corresponding LSVs are utilized to estimate the angle space correlation between the training and test data. Moreover, the singular vectors are allocated with different weights according to their corresponding singular values. The ds-HD is used to compute the scatterer distance and the matching degree between the training and test data. Finally, the results of both numerical experiments and real measured outfield experiments are provided, which demonstrate the effectiveness and robustness of the proposed SSM-ANM method.

In our future work, we will explore the situation that the target is occluded, where the performance of the SSM-ANM method will be affected, especially when the occlusion is severe. We will make further improvement to increase the robustness to occlusion by adapting the weight of the occluded scatterers in recognition. Besides, the open set recognition problem will be considered if the object is exclusive in the training data. Thus, a classifier with rejection capability needs to be exploited.

## Figures and Tables

**Table 1:** Algorithmic recap of the SSM-ANM method.

**Algorithmic Recap of the SSM-ANM Method**
**Input:** The training data ${\left\{X_{N\times M_{d,g}}^{d.g}\right\}}_{d=1,g=1}^{D_{g}\;G}$ with G types of targets and $D_{g}$ aspect-frames for gth target. The test data $Y_{N\times M}$. Get the SVD result for $Y_{N\times M}$, denoted by $Y_{N\times M}=U^{0}\Lambda^{0}V^{0}$. Choose the *t* dominant singular values and singular vectors, represented as $\left\{\lambda_{1}^{0},\lambda_{2}^{0},\cdots ,\lambda_{t}^{0}\right\}$, $\left\{u_{1}^{0},\cdots ,u_{t}^{0}\right\}$ and $\left\{v_{1}^{0},\cdots ,v_{t}^{0}\right\}$. Extract the location and intensity information of the dominant scatterers for $v_{i}^{0}\left(i=1,\cdots ,t\right)$. Calculate the matching score $o^{d.g}$ for each training data $X_{N\times M_{d,g}}^{d.g}$. For *g* = 1 to G For *d* = 1 to $D_{g}$ Get the SVD result for $X_{N\times M_{d,g}}^{d.g}$, denoted by $X_{N\times M_{d,g}}^{d.g}=U^{d,g}\Lambda^{d,g}V^{d,g}$. Choose the *s* dominant singular values and singular vectors, represented as $\left\{\lambda_{1}^{d,g},\lambda_{2}^{d,g},\cdots ,\lambda_{s}^{d,g}\right\}$, $\left\{u_{1}^{d,g},\cdots ,u_{s}^{d,g}\right\}$ and $\left\{v_{1}^{d,g},\cdots ,v_{s}^{d,g}\right\}$. Extract the location and intensity information of the dominant scatterers by ANM for $v_{j}^{d,g}\left(j=1,\cdots ,s\right)$, as represented by ℙ. Calculate the ds-HD for $v_{i}^{0}$ and $v_{j}^{d,g}$ for each i=1,⋯,t and j=1,⋯,s, and form the matching matrix $M_{s\times t}^{d,g}$. Calculate the correlation for $u_{i}^{0}$ and $u_{j}^{d,g}$ for each i=1,⋯,t and j=1,⋯,s, and denoted by a correlation matrix $Z_{s\times t}^{d.g}$. Get the matching score $o^{d.g}$ according to the following definition (15) **End** **End** Find the largest score $o^{d_{0}.g_{0}}$ corresponding to the type $g_{0}$ with aspect-frame $d_{0}$. **Output:** The recognition result $g_{0}$ for the test data $Y_{N\times M}$.

**Table 2:** Relative radial range and intensity for the scatterers.

Scatterer Number	A	B	C	D	E	F
Radial range/m	3.28	2.05	0.41	−0.82	−2.05	−3.27
Intensity	0.3	0.3	1	1	0.2	0.2

**Table 3:** Simulation system parameters.

Parameter	Value
Carrier frequency $f_{0}$	X-band
Waveform	Chirp
Bandwidth B	1 GHz
Pulse repetition frequency	50 Hz
Pulse width $T_{p}$	2 ms
CPI	10 pulses

**Table 4:** Matching scores comparison of the five targets by $O_{r}^{d,g}$ and $O^{d,g}$.

Training HRRPs	**Test HRRPs**				
		Type A	Type B	Type C	Type D
	Type A	1.00/**1.00**	0.58/**0.24**	0.17/**0.04**	0.43/**0.05**
	Type B	0.59/**0.47**	1.00/**1.00**	0.41/**0.18**	0.75/**0.30**
	Type C	0.16/**0.13**	0.53/**0.27**	1.00/**1.00**	0.52/**0.19**
	Type D	0.35/**0.10**	0.81/**0.14**	0.29/**0.04**	1.00/**1.00**

**Table 5:** Computational complexity of different methods.

Methods	Computational Complexity
SSM-ANM	O(MN^2^) + O(M^3^) + O(N^3^)
SSM-OMP	O(MN^2^) + O(MK^2^) + O(N^3^)
HSM-OMP	O(MK^2^)
RSM-ANM	O(N^2^M) + O(M^3^)

**Table 6:** Real data recognition result for the SSM-ANM method.

	Target 1	Target 2	Target 3	Target 4
**Target 1**	**0.81**	0.04	0.06	0.09
**Target 2**	0.01	**0.87**	0.05	0.07
**Target 3**	0.04	0.02	**0.83**	0.11
**Target 4**	0.13	0.01	0.01	**0.85**
Average recognition rate: 0.84				

**Table 7:** Real data recognition result for the SSM-OMP method.

	Target 1	Target 2	Target 3	Target 4
**Target 1**	**0.72**	0.06	0.09	0.13
**Target 2**	0.03	**0.81**	0.05	0.11
**Target 3**	0.08	0.04	**0.76**	0.12
**Target 4**	0.16	0.03	0.07	**0.74**
Average recognition rate: 0.76				

**Table 8:** Real data recognition result for the SSM-OMP method without LSVs.

	Target 1	Target 2	Target 3	Target 4
**Target 1**	**0.76**	0.04	0.07	0.13
**Target 2**	0.01	**0.83**	0.06	0.10
**Target 3**	0.06	0.05	**0.75**	0.14
**Target 4**	0.12	0.07	0.03	**0.78**
Average recognition rate: 0.78				

**Table 9:** Real data recognition result for the HSM-OMP method.

	Target 1	Target 2	Target 3	Target 4
**Target 1**	**0.68**	0.08	0.11	0.13
**Target 2**	0.06	**0.72**	0.10	0.12
**Target 3**	0.08	0.07	**0.70**	0.15
**Target 4**	0.21	0.06	0.08	**0.65**
Average recognition rate: 0.69				
